# Experimental validation of a predicted microRNA within human *FVIII *gene

**DOI:** 10.22099/mbrc.2021.39067.1573

**Published:** 2021-06

**Authors:** Shahabeddin Jalali-Qomi, Majid Motovali-bashi, Halimeh Rezaei, Sheyda Khalilian

**Affiliations:** Department of Cell and Molecular Biology and Microbiology, Faculty of Biological Science and Technology, University of Isfahan, Iran

**Keywords:** Hemophilia A, Factor VIII, Small non-coding RNA, miRNAs, HEK 293 cell line

## Abstract

Hemophilia A is an X-linked bleeding disorder that occurs due to the deficiency of Factor VIII (FVIII) protein clotting activity. The mutations in the *F8* gene, which encodes FVIII coagulating protein have been widely reviewed. However, there is a wide range of criteria that in addition to *F8* gene mutations, different molecular mechanisms may be associated with hemophilia A. Various functions of FVIII could be related to the hypothetical small non-coding RNAs, located within the *F8* gene sequence. Therefore, miRNAs that can post-transcriptionally regulate gene expression might confer susceptibility to developing hemophilia A. Here, we have selected a bioinformatically predicted hairpin structure sequence in the first intron of the *F8* gene that has the potential to produce a real miRNA (named put-miR1). We tried to experimentally detect the predicted miRNA via RT-PCR following its precursor overexpression in HEK 293 cell lines. Despite the accuracy of miRNA prediction, according to the reliable bioinformatics studies, we couldn’t confirm the existence of considered mature miRNA in transfected cells. We hope that through changing experimental conditions, designing new primers, or altering cell lines and expression vectors, the exogenous and endogenous expression of the predicted miRNA will be confirmed.

## INTRODUCTION

Hemophilia A is a coagulation defect caused by *F8* gene mutations and leads to the production of an abnormal coagulating Factor VIII (FVIII) [1, 2]. The inactive form of FVIII is secreted into the plasma and attaches to the Von Willebrand factor (vWF) to make a complex that prevents its degradation. Activated FVIII, in response to injury, detaches from vWF and takes part in formation of the clotting cascade [3]. Hemophilia A can be effectively managed with continuous infusions of the FVIII, to eradicate FVIII inhibitors and restore normal FVIII [4]. These days developing inhibitors is an important treatment complication, which occurs in about 25-30% of patients [5]. *F8* gene certain mutations and the risk of developing inhibitors have leads to the studies that largely focused on the *F8* gene deficiencies [6]. More than one thousand various mutations have been identified in the *F8* gene, including inversions, substitutions, deletions, etc. [7]. Furthermore, different mechanisms such as *F8* gene inhibition can regulate the severity or progression of the disease [8, 9].

Previously, non-coding RNAs (ncRNAs) were known as key regulators of gene expression [10]. Recently, the involvement of small non-coding RNAs (sncRNAs) like microRNAs (miRNAs) in complicated biological processes has been clarified, and ncRNA defects have been reported in many human diseases [11]. In this respect, miRNAs represent the most explored sncRNA species in humans, supported by a large number of publications [12]. miRNAs are categorized as a class of ncRNAs, 18-25 nucleotide-long that control the expression of target genes, as a component of complex gene regulatory networks, and can regulate some biological pathways [13].

The miRNA precursor (pre-miRNA) secondary structure is rapidly processed into a pre-miRNA molecule with a hairpin structure. The pre-miRNA is then transported to the cytoplasm and further processed into miRNA mature form, which pairs with its target transcripts and regulates their function [14]. Although previous researches suggest that there may be about 55,000 miRNA genes throughout the human genome [15], there are now over 17,000 distinct mature miRNAs that have been discovered in over 140 species [16]. Thus, various bioinformatics tools and databases have been devised to predict novel miRNAs [17]. Briefly, some characteristics such as precursor secondary structure, evolutionary conservation, minimal folding free energy, etc. are used in the computational identification of novel miRNAs [18]. 

Since hemophilia A is a single-gene hereditary disorder, we decided to select the *F8* gene for our genomic analysis. Herein, we have selected put-miR1, a bioinformatically predicted hairpin structure sequence in the first intron of the *F8* gene, that has the features of producing a mature miRNA, and examined its overexpression in HEK 293 cells.

## MATERIALS AND METHODS

**Bioinformatics studies: **A bioinformatically predicted intronic hairpin structure sequence (put-miR1), that was previously reported in our research [19] was selected for subsequent validation. In order to predict this hairpin structure within the area of interest, examine its conservation, and also evaluate the existence of similar sequences, the SSC Profiler (http:// mirna.imbb.forth.gr/SSCprofiler.html), miREval (http://mimirna.centenary.org.au/mireval/), FOMmiR (http://app.shenwei.me/cgi-bin/FOMmiR.cgi), RNAfold algorithm (http://rna.tbi. univie.ac.at/cgi-bin/RNAfold.cgi), UCSC database (http://genome.ucsc.edu/), miR-FIND (http: //140.120.14.132:8080/MicroRNAProject-Web/), and finally, miRBase databases (http://www. mirbase.org/index.shtml) were used by authors and explained in detail in the cited article.

**DNA preparation: **The isolation of the genomic DNA from human whole blood was performed through a salting out procedure [20]. The candidate region in the *F8* gene that can express the possible miRNA secondary structure, was PCR amplified using primers designed by Oligo v.7 and PerlPrimer (Table 1), using the following conditions: Initial denaturation: 94°C for 5 min, Denaturation: 35 cycles at 94°C for 30 s, Annealing: 56°C for 30 s, Extension: 72°C for 30 s, Final extension: 72°C for 10 min. Gel electrophoresis was carried out to examine the PCR product's specificity. Using the Gel Extraction Kit (GeNetBio/Korea), DNA samples were purified and cloned into pTZ57R/T (Thermo scientific/USA) and then transformed into *Escherichia coli TOP10 strains *using a standard protocol [21]. The transformed cells with 75 mg mL^-1^ ampicillin and 20 mg mL^-1^ ×-Gal were then plated on the LB agar. Colonies were randomly selected and colony PCR was then performed using the TA vector primer pair, designed by Oligo v.7 (Table 1), and also the primer pairs of input DNA fragment previously described. Purification of plasmid DNA from bacterial DNA was carried out using PrimePrep Plasmid DNA Isolation Kit (GeNetBio/Korea). Recombinant TA vectors were digested by *KpnI* and *SacI* restriction enzymes (in 37°C for 10 min) and cloned into the double digested pEGFP-C1 expression vector with the same restriction enzymes, downstream of the GFP sequence. Then, transformation of the competent cells of *Escherichia coli* *DH5-α* strains, was performed by pEGFP-C1 recombinant vector, and in order to validate the correctness of transformation process colony PCR was carried out. Finally, in order to verify the inserted fragment, vectors were sequenced (Genfanavaran Co.).

**Table 1:** List of primers used in the present study

**Primer**	**Sequence (5′‐3′)**	**Usage**
**put-miR1 precursor**	F: AAGCAGAGAGACTAACAGGT	Amplification of the gene fragment containing
	R: GAGGCTGCTAAACTTAACCC	the miRNA precursor
**TA vector**	F: GTAAAACGACGGCCAGT	Cloning
	R: CAGGAAACAGCTATGAC	
**Mature put-miR1**	F1: GCCACTCAGGAAGAGGGTTG	cDNA synthesis
	F2: CTCAGGAAGAGGGTTGGAG	
	F3: AGGAAGAGGGTTGGAGTAGG	
	F4: GAGGGTTGGAGTAGGCTAGG	
	F5: GGTTGGAGTAGGCTAGGAAT	
	F6: TTGGAGTAGGCTAGGAATAGG	
	F7: GGAGTAGGCTAGGAATAGGAG	
	F8: GTAGGCTAGGAATAGGAGCAC	
	F9: GAGCACAAATTAAAGCTCCTG	
	F10: AAGCTCCTGTTCACTTTGAC	

* Reverse primers for Mature Put-miR1 are provided by the cDNA synthesis kit (universal primers)

**Cell Line: **HEK 293 cells were grown in DMEM-HG basal medium (Invitrogen) supplemented with 100 U/mL penicillin, 100 µg/mL streptomycin, and 10% FBS, and transfected with pEGFP-C1 recombinant vector by the standard calcium phosphate method [24]. Cultures utilizing un-transfected cells (to confirm the accuracy of the transfection), mock-transfected cells (to confirm the validity of the vector insertion) and scramble-transfected cells (to confirm the accuracy of the cell line-inserted fragment) were used as the negative controls.

**RNA Extraction:** Total RNA extraction from HEK 293 cells was performed using Trizol reagent based on the manufacturer’s summary instructions (SIGMA). Treatment with RNAase free DNAaseI (Takara) at 37ºC for 30 min was carried out following with 10 min heat inactivation at 65ºC by adding EDTA. The extracted RNA was qualified on 2% agarose gel. Furthermore, RNA concentration and purity were assessed by NanoDrop.

**cDNA synthesis and PCR amplification:** Using the Universal cDNA Synthesis Kit II (Exiqon/MA/USA), cDNA synthesis was carried out. About 0.1 ug of isolated RNA in the total volume of 20 μL were reverse transcribed, using oligo-dT primers, incubated for 1h at 42°C followed by 5 min enzyme thermal inactivation at 95°C. The whole mixture was used for PCR to confirm the expression of mature miRNAs. PCR was performed using Taq DNA Polymerase 2X Master Mix Red (Ampliqon, Denmark), specific designed primers and also oligo-dT primers provided by cDNA Synthesis kit, in the total volume of 20 μL according to the manufacturer^'^s protocol. Amplification was carried out using the following conditions: A single cycle at 94°C for 5 min, 38 cycles at 94°C for 30 s, 60°C for 30 s, and 72°C for 30 s. At last, the final extension was performed at 72°C for 10 min. Finally, the PCR products were qualified on 12% polyacrylamide gel.

## RESULTS

In our previous study, reliable bioinformatics databases were employed due to elicit candidate stem-loops within the human *F8* gene that express miRNA. Eventually, Two putative miRNA precursors were predicted and presented for experimental validation. Based on the UCSC genome browser, the miRNA precursors were conserved in mammals and no similar miRNA sequence has been reported in the miRBase database [19]. In the present study, we have selected put-miR1, with the sequence of “TGC TGC TGC CAC TCA GGA AGA GGG TTG GAG TAG GCT AGG AAT AGG AGC ACA AAT TAA AGC TCC TGT TCA CTT TGA CTT CTC CAT CCC TCT CCT CCT TTC CTT AA”, located in the first intron of the *F8* gene to be validated experimentally. 

**Figure 1:**
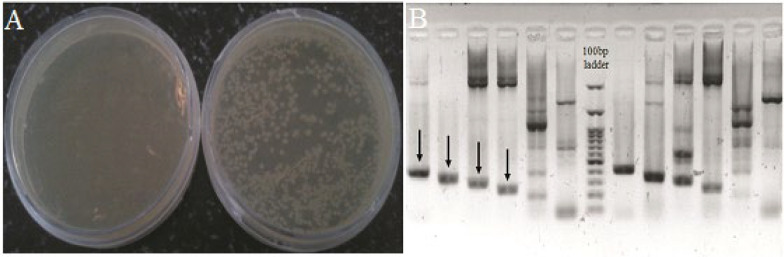
Results of transformation and colony PCR. (A) Transformation of recombinant TA vector, containing predicted pre-miRNA sequences, into TOP10 strains. Right: positive control, left: negative control. (B) Colony PCR for colonies containing Candidate miR and recombinant TA vector, respectively. Staging bands that confirm the insertion accuracy of the fragments are indicated by arrows

After genomic DNA extraction from whole blood, the mean A_260_/A_280_ nm ratio was calculated by NanoDrop ND-1000 (NanoDrop Tech) to assess the purity of the DNA. The ratios were ranged consistently from 1.8 to 2.0, which demonstrated a good deproteinization. Furthermore, the genomic DNA integrity was determined using 1% agarose gel electrophoresis. The genomic regions containing a sequence of 104 nucleotides putative miRNA precursors were PCR amplified by specific primers under conditions described before. Additionally, the specification test for the PCR products was performed via 1% agarose gel electrophoresis. The expected amplification fragments in 256 bp containing predicted miRNA precursors, were purified and cloned in the TA vector pTZ57R/T. Then, transformation was carried out in the *Escherichia coli* TOP10 strains and cultured in LB agar amended with ampicillin and x-Gal. Also, the recombinant TA vector was cultured on another plate under the same conditions as a negative control (Fig. 1A). Transformed colonies were verified by colony PCR (Fig. 1B). The PCR products were separated via agarose gel electrophoresis, and the presence of expected bands verified the correct insertion. Afterward, the amplified products were extracted from the gel and double digested with *Kpn*I and *Sac*I, in order to be cloned into the pEGFP-C1 expression vector at the same restriction sites (Supplementary Fig. S1). 

**Figure 2:**
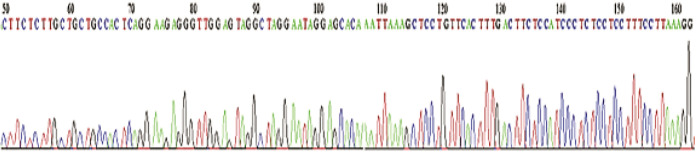
Sequencing results. Outputs approve the sequence accuracy of inserts, respectively

The pEGFP-C1 recombinant expression vector was transformed into the component cells of *Escherichia coli* DH5-α strain and plated on LB agar amended with kanamycin. Colony PCR was performed to screen positive colonies and agarose gel electrophoresis confirmed this purpose. In order to final confirmation of recombinant expression vector, double digestion of the vector by mentioned restriction enzymes was carried out, and the products were analyzed by electrophoresis. The presence of expected bands could validate the correct insertion. Finally, recombinant vectors sequencing was carried out to approve the sequence accuracy of inserts, which demonstrated a complete homology between the inserted fragment and the predicted miRNA precursor (Fig. 2).

**Figure 3:**
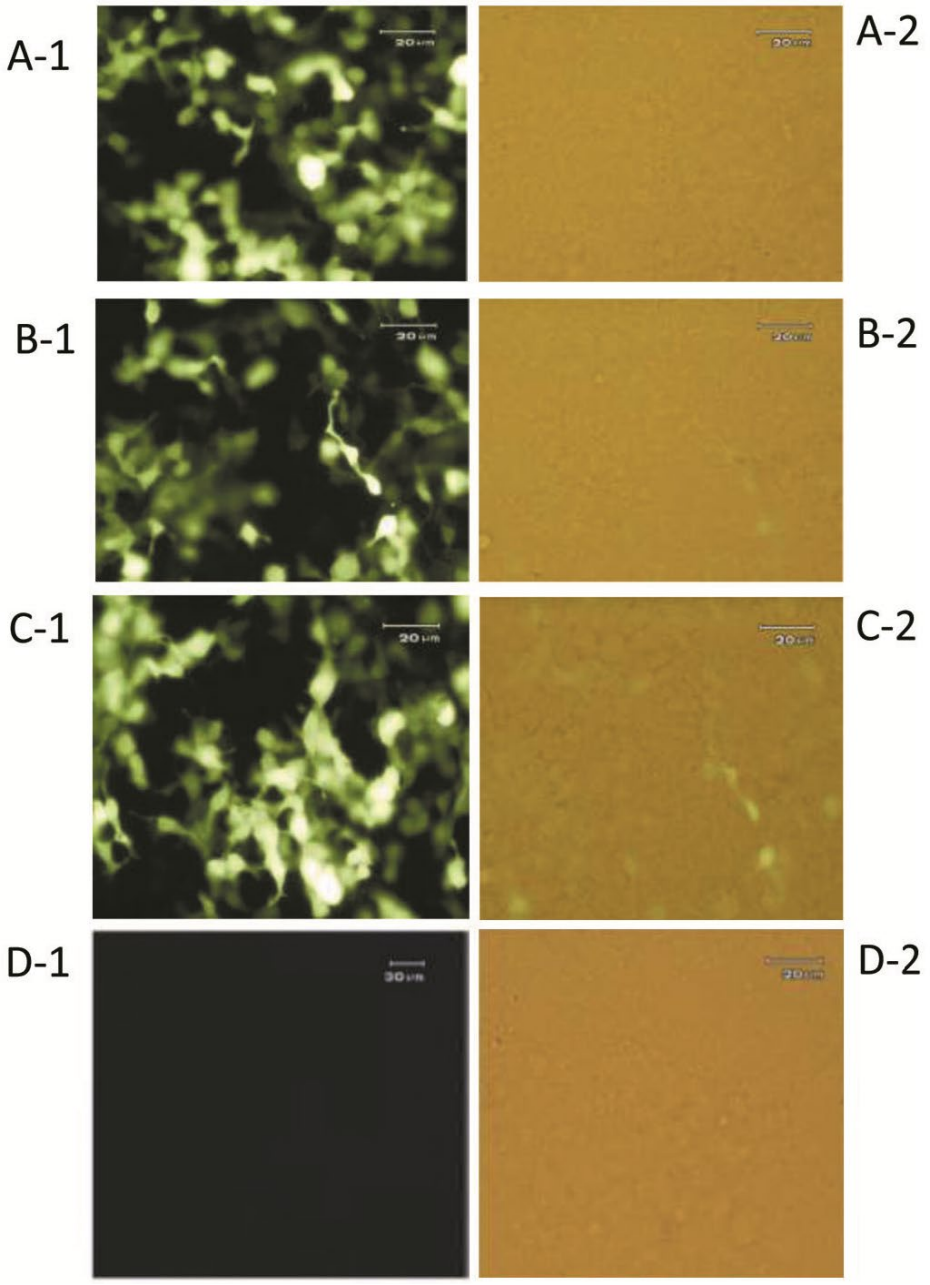
GFP protein expression using florescence microscopy. GFP expression indicates transfection accuracy in (A-1, A-2) Pre-miRNA, (B-1, B-2) Scramble, (C-1, C-2) Mock and (D-1, D-2) untransfected HEK 293 cell line

To ensure transfection efficiency, GFP expression was visualized via fluorescence microscopy 36 hours after HEK 293 cell line transfection (Fig. 3). About, 48 hours after transfection, total RNA was extracted. By measuring the samples concentration using a spectrophotometer, and loading 3μl of the samples on 2% agarose gel, the quantity and the quality of RNAs were assessed, respectively. Observation of the 28S and 18S ribosomal RNA bands at the top and the 5S and 5.8S bands at the bottom of the agarose gel indicated that the extracted RNA samples are undamaged and of good quality. The average concentration of extracted RNAs was 400 ng/μl.

**Figure 4:**
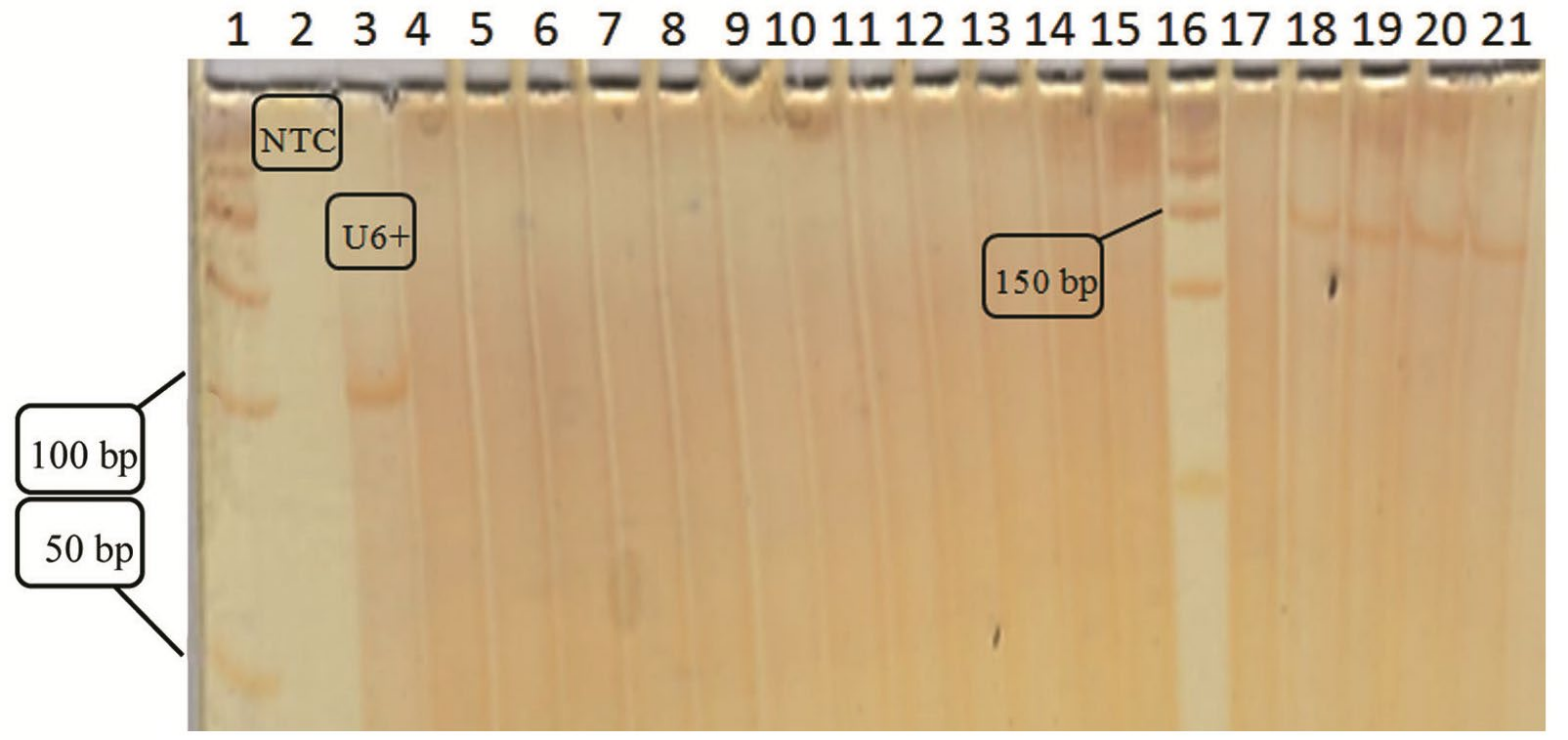
Polyacrylamide gel electrophoresis to confirm the mature miRNA expression. Wells related to the negative control (NTC) and positive control (U6+) are indicated on the figure. The subsequent wells are related to the PCR products, using primers designed for the candidate mature miRNA at different annealing temperatures

After cDNA synthesis, to confirm the presence of mature miRNA, each cDNA was amplified in a PCR system. Several primers were designed according to the sequence recommended by bioinformatics servers and used as the forward primer (Table 1). The universal primers provided by the cDNA synthesis kit were used as the reverse primer. After PCR, due to the small size of the fragments (80-100 bp), using U6RNA as a reference gene, PCR products were loaded on 12% polyacrylamide gel. Unfortunately, no bands of any kind of primer were seen and we couldn’t confirm the existence of considered mature miRNA in transfected cells (Fig. 4).

## DISCUSSION

By discovering miRNAs and proving their significant function in regulating gene expression, researchers have found increasing evidence based on aberrant expression of some miRNAs in various disease [23-25]. In this regard, several bioinformatics tools and computational algorithms have been devised to help the efficient prediction of novel miRNAs in genomic sequences [17, 26]. So far, most of the miRNA identification has been accomplished by RNA cloning and sequencing. Various protocols have been developed for this purpose and successfully used to identify most of the currently detected miRNAs [27, 28]. 

Recently, Rahaimi et al, experimentally investigated the existence of a predicted miRNA in breast cancer cells. They used SSC profiler, RNAfold, miRNAFold, MiPred, and FOMmiR bioinformatics tools to investigate potential hairpin structures within human CDH4 gene and identify true miRNA precursors. The hsa‐miR‐B43 was finally selected as the candidate for in vitro validation. They could detect the exogenous and endogenous hsa‐miR‐B43 expression in cancer cell lines, and indicated hsa‐miR‐B43, as a novel miRNA, which could have a function in the metastatic process [29].

Hoballa et al, using the bioinformatics tools, introduced miRZa-3p and miRZa-5p as two novel miRNAs, which can target IGF1R and SMAD3 [30]. Additionally, in a similar fashion Dokanehiifard et al, predicted a miRNA called TrKC-miR2, located in the TrKC gene, as a novel Wnt pathway regulator that could be a candidate biomarker for colorectal cancer [31]. Medlej et al, predicted a stem‐loop structure located in the *GATA4* gene, and detected its exogenous and endogenous expression. Besides, they introduced IGF‐1R and AKT1/2 genes as potential targets for GATA4‐miR1 via in silico analysis [32]. 

In a broad study, Wake et al, devised a method utilizing miRDeep to explore novel miRNAs from concatenated small RNA sequencing samples. They identified 99 potential miRNAs in human brain tissues, and seven of them were experimentally validated using qPCR [33]. Furthermore, in 2018, Gottmann et al, applied a computational framework using transcriptomics and miRNA prediction tools to elevate the potential of miRNA discovery. Therefore, they could identify novel miRNAs related to type 2 diabetes and obesity [34].

According to the wide range of researches, there is mounting criteria that ncRNAs might have an important function in the FVIII deficiency in hemophilia A disease. Sarachana et al, performed a microarray analysis on several hemophilia A patients to examine the hypothesis that miRNAs up-regulation or down-regulation has been associated with the pathology of hemophilia A. Their results showed that the expression level of miR-4521, miR-1246, and miR-181d in the hemophilia A patients increased significantly [35]. In another study, the role of miRNAs in inhibiting FVIII in hemophilia A patients was investigated using NGS. Data analysis indicated the decreased level of miR-128-3p and let-7i-5p and increased level of miR-144-5p, miR-374b-5p, miR-30c-5p, miR-6803-3p, miR-15b-3p and miR-483-3p in the patients [36]. These studies confirmed miRNAs important role in regulating the FVIII levels. Besides, increasing evidence showed that miRNA dysregulation has been associated with FVIII deficiency, and could suggest a possible method for the therapeutic application of miRNAs in Hemophilia A treatment [37].  

While cloning and sequencing were used to identify most of the novel miRNAs, increasing efforts are being put into bioinformatics prediction of new miRNAs [38, 39]. In the present study, the *F8* human genomic region was scanned to investigating the presence of hairpin stem-loop structures, using bioinformatics tools. For this purpose, the predicted stem-loop sequence was cloned into an expression vector and the presence of the precursor mature form was examined in HEK 293 cells.

The criteria for pri-miRNA prediction was strongly supported by reliable bioinformatics studies, but we could not detect the candidate miRNA in the transfected cell lines. It seems that through changing experimental conditions or altering cell lines and expression vectors, the exogenous and endogenous expression of the predicted miRNA will be confirmed. For instance, utilizing primary hepatic cells or Huh7 or HepH2 liver cells would be probably informative. Additionally, changing PCR conditions and cDNA synthesis method or designing new primers are another probable thesis to detect the candidate miRNA in transfected cell lines. Deep exploration is suggested to start from some classical and known miRNAs in addition to the experimental conditions. Pathway analysis is also recommended to perform, through systems biology approach, in order to raise our knowledge about the relevant biological functions controlled by the miRNA signature. 

Therefore, more researches are required to confirm the presence of this candidate miRNA, in the light of the above points. After confirmation of the miRNA at this stage, the expression levels should be measured within the transfected and non-transfected cell populations using Real time-PCR. Then we can review and compare the miRNA expression between affected and healthy people.

## Supplementary Materials

Supplementary Fig. S1
