# Supplementary material for: Experimental validation of a predicted microRNA within human FVIII gene
Source: Mol Biol Res Commun. 2021 Jun;10(2):45–53. doi: 10.22099/mbrc.2021.39067.1573 (PMC8310658; doi:10.22099/mbrc.2021.39067.1573)
Supplement: Supplementary Fig. S1 [file mbrc-10-45-s001.pdf]

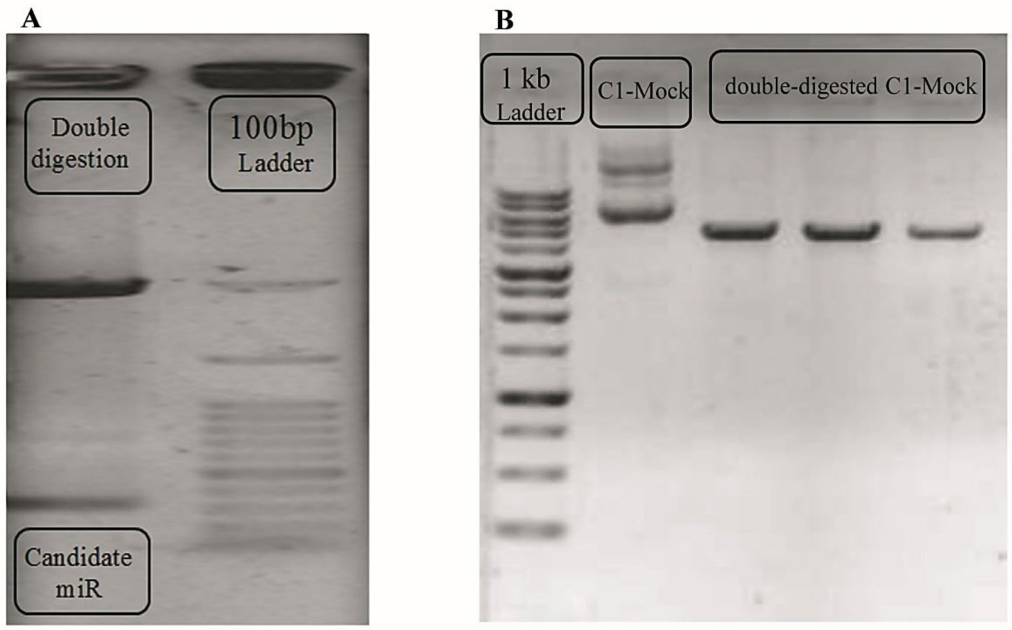

Figure S1. Double digested (*KpnI*/*SacI*) samples gel electrophoresis. (A) Gel electrophoresis of double digested recombinant TA vectors. The linearized TA vector band after double digestion and also the band related to the fragment containing miRNA precursor is observed, respectively. (B) Gel electrophoresis of double digested pEGFP-C1 vector. The single band pattern for digested vector and multi-band pattern for C1-Mock is shown.
